# miR-199b, a novel tumor suppressor miRNA in acute myeloid leukemia with prognostic implications

**DOI:** 10.1186/s40164-016-0033-6

**Published:** 2016-02-03

**Authors:** Amanda J. Favreau, Rose E. McGlauflin, Christine W. Duarte, Pradeep Sathyanarayana

**Affiliations:** 1 Center for Molecular Medicine, Maine Medical Center Research Institute, 81 Research Drive, Scarborough, ME 04074 USA; 2 The Graduate School of Biomedical Science and Engineering, University of Maine, Orono, ME 04469 USA; 3 Department of Medicine, Tufts University School of Medicine, Boston, MA USA

**Keywords:** Acute myeloid leukemia, miRNA-199b, HSC, FAB-M5, NPM1, Bone marrow transplant

## Abstract

**Background:**

Dysregulation of miRNAs that can act as tumor suppressors or oncogenes can result in tumorigenesis. Previously we demonstrated that miR-199b was significantly downregulated in acute myeloid leukemia (AML) and targets podocalyxin and discoidin domain receptor 1. Herein we investigated the functional role of miR-199b in AML and its prognostic implications.

**Methods:**

Major approaches include transduction of hematopoietic stem cells and bone marrow transplantation, analyses of blood lineages, histone deacetylases (HDAC) inhibitors, and molecular and clinical data analyses of AML patients using The Cancer Genome Atlas (TCGA).

**Results:**

We first examined the relative miR-199b expression in steady state hematopoiesis and showed CD33^+^ myeloid progenitors had the highest miR-199b expression. Further, silencing of miR-199b in CD34^+^ cells resulted in significant increases in CFU-GM colonies. Via TCGA we analyzed the molecular and clinical characteristics of 166 AML cases to investigate a prognostic role for miR-199b. The Kaplan–Meier curves for high and low expression values of miR-199b and the observed distribution of miRNA expression revealed the highly expressed group had significantly better survival outcomes (p < 0.016, log rank test). Additionally, there was significant difference between miR-199b expression across the AML subtypes with particularly low expression found in the FAB-M5 subtype. Furthermore, FAB-M5 subtype showed a poor prognosis with a 1-year survival rate of only 25 %, compared with 51 % survival in the overall sample (p < 0.024). Furthermore, significant inverse correlation of HoxA7 and HoxB6 expression with miR-199b was observed in FAB-M5 AML patients. Molecular mutations were analyzed among miR-199b high and low AML cases. Significant correlations in terms of association and survival outcomes were observed for NPMc and IDH1 mutations. Treatment of THP-1 cells (represents M5-subtype) with HDAC inhibitors AR-42, Panobinostat, or Decitabine showed miR-199b expression was significantly elevated upon AR-42 and Panobinostat treatment. To further understand the hematopathological consequences of decreased miR-199b, we employed a bone-marrow transduce/transplant (BMT) mouse model. Interestingly, in vivo miR-199b silencing per-se in HSCs did not result in profound perturbations.

**Conclusions:**

Loss of miR-199b can lead to myeloproliferation while HDAC inhibitors restore miR-199b expression and promote apoptosis. Low miR-199b in AML patients correlates with worse overall survival and has prognostic significance for FAB-M5 subtype.

**Electronic supplementary material:**

The online version of this article (doi:10.1186/s40164-016-0033-6) contains supplementary material, which is available to authorized users.

## Background

**Fig. 1:**
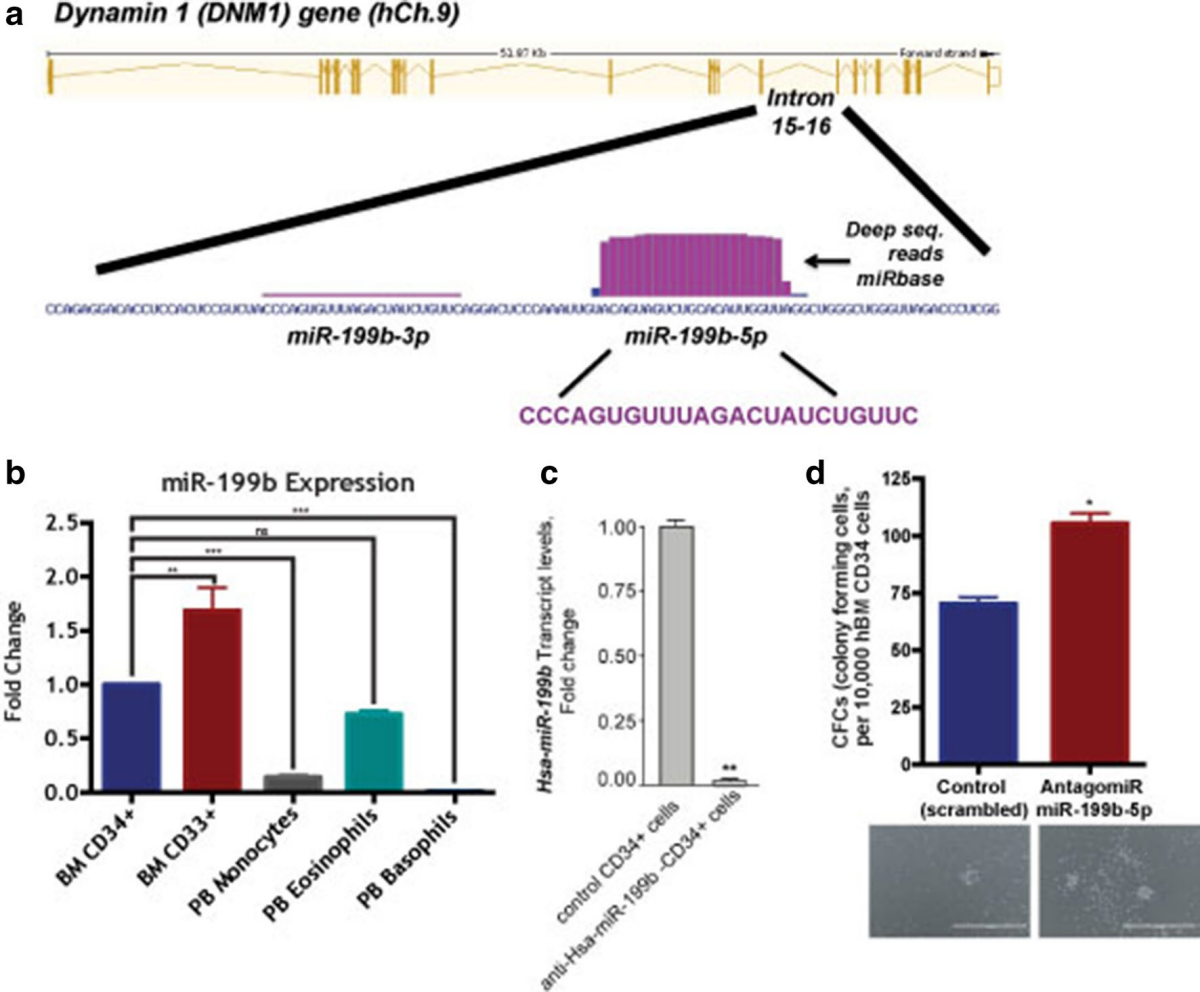
Intronic miR-199b expression in steady state hematopoiesis and its myelopoietic and hematopoietic potential. **a** miR-199b’s location within the genome; intronic of the DNM1 gene. **b** Human primary BM CD34^+^, BM CD33^+^, PB monocytes, PB eosinophils, and PB basophils expression of miR-199b in steady state hematopoiesis. **c** Lentiviral particles for anti-Has-miR-199b-5p and control vector were used to transduce human BM derived CD34 + cells. Silencing of miR-199b levels were confirmed in HSCs post transduction via RT-qPCR. **d** Lentiviral particles for anti-hsa-miR-199b-5p and scrambled/control vector were used to transduce human BM derived CD34 + cells. CFU-GM assay was performed and representative photomicrographs of the colonies are shown

Acute myeloid leukemia (AML) manifests a marked heterogeneity in both response to therapy and patient survival, observations that likely reflect its varied pathogenesis [1]. Dysregulation of miRNAs that can act as tumor suppressors or oncogenes can result in tumorigenesis. One miRNA of interest is human miR-199b-5p. Human miR-199b-5p is located on chromosome 9 and is an intragenic miRNA encoded in the dynamin 1 (DNM1) gene, from the opposite strand in a 2.2 kb intronic region between exons 14 and 15 (Fig. 1a). Mature 5p arm, encoded by hsa-miR-199b, is evolutionarily conserved among several species. Transcription of miR-199b is predicted to be independent of its host gene, DNM1. Studies to date have demonstrated that miR-199b targets nuclear kinase Dyrk1a and promotes calcineurin/NFAT signaling in cardiomyocytes. Suppression of miR-199b expression has been reported in hepatocellular carcinoma and predicted poor prognosis in this disease [2]. Further, miR-199b was demonstrated to target HIF-1alpha in hepatocarcinoma cells and prostate cancer cells [2, 3]. miR-199b has also been showed to target HER2 in breast cancer cells [4]. Notably, a recent investigation revealed overexpression of miR-199b-5p in medulloblastoma cancer stem cells results in downregulation of CD133^+^ tumor initiating cells and causes depletion of the side population compartment of tumor stem cells via negative regulation of the Hes1-mediated Notch signaling pathway [5].

Previously, we demonstrated miR-199b was significantly downregulated in AML and targets podocalyxin and discoidin domain receptor 1 (DDR1) to regulate migration [6]. Down-regulation of miR-199b has been strongly associated with imatinib drug resistance in 9q34.1 deleted BCR/ABL positive CML patients [7]. Silencing of miR-199b has also been associated with acquired chemoresistance in ovarian cancer, possibly mediated via activation of JAG-Notch1 signaling pathway [8]. Interestingly, successful delivery of miR-199b in tumorigenic cell lines via stable nucleic acid particles (SNALPs) lead to impaired cell proliferation, however, no apoptosis was observed [9]. MiR-199 locus has also been implicated in intracellular trafficking where miR-199b and miR-199a together has been demonstrated to control receptor-mediated endocytosis via inhibiting CLTC, Rab5A, LDLR and Cav-1 expression [10].

The cause of miR-199b-5p downregulation in AML has yet to be examined. Studies have revealed 20 % of individuals with AML harbor somatic mutations in DNMT3A and genome-wide sequencing approaches have strengthened the notion that AML can be classified on the basis of patterns of promoter methylation [11, 12]. We hypothesized that future investigations into the epigenetic status such as histone modification via histone deacetylases (HDACs) or DNA methylation of miR-199b-5p’s promoter may further unravel the link between aberrant epigenetic regulation and subsequent pathogeneic consequences manifested via the dysregulated expression of miR-199b-5p’s target genes as in silico analysis shows five upstream CpG islands (data not shown). Furthermore, miR-199b expression in steady state hematopoiesis has yet to be analyzed.

By gaining better insight into miR-199b-5p’s expression in steady state hematopoiesis, we can investigate the prognositic consequences of miR-199b-5p’s loss in depth in AML as well as determine if it can become a therapeutic target for specific subtypes of AML. Herein, we aim to determine the hematopoietic/myelopoietic consequences of miR-199b silencing ex vivo and in vivo via BM transplantation approach, clinical significance especially prognostic significance in AML and epigenetic regulation of miR-199b-5p in AML to garner more insight into its therapeutic potential. Additionally, in vivo investigations involving combinatorial deletions of cooperative factors like NPM1 or IDH1/2 are required to determine if miR-199b has leukemogenic potential. This will elucidate if loss of miR-199b-5p directly contributes to leukemogenesis or if it is better served as a diagnostic and prognostic marker.

## Results and discussion

Previously, we demonstrated miR-199b-5p was downregulated in a subset of AML patients but didn’t have sufficient clinical details about the patients to draw a distinct correlation to miR-199b expression and AML subtype or overall survival. Additionally, both miR-199b’s normal expression pattern in hematopoiesis and its regulation have yet to be examined. Our current studies investigate the role of miR-199b in normal hematopoiesis and AML utilizing both ex vivo and in vivo approaches and analyzing prognostic data obtained from The Cancer Genome Atlas (TCGA) on miR-199b expression in AML patients.

### miR-199b in normal hematopoiesis

To begin, we examined relative miR-199b expression in steady state hematopoiesis and showed CD33^+^ myeloid progenitors had the highest miR-199b expression, which drops upon differentiation (Fig. 1b). To test if suppression of miR-199b results in myeloproliferation and HSC proliferation, human bone marrow derived CD34^+^ cells were transduced with anti-miR-199b or control lentivirus a CFU assay respectively. Silencing of miR-199b expression was confirmed via RTqPCR (Fig. 1c). Further, a functional validation of miR-199b silencing was tested by examining the levels of HIF1-alpha, a known and validated target of miR-199b [2]. Transcript levels of HIF1-alpha were significantly elevated in hCD34 cells expressing anti-miR-199b (Additional file 1: Figure S1). At day 16, silencing of miR-199b in CD34^+^ cells resulted in significant increases in CFU-GM colonies strongly suggesting dysregulation of miR-199b expression has a role in myelopoiesis and may be leukemogenic (Fig. 1d). The relatively low values observed for CFU-GM is possibly arising from the usage of cryopreserved human BM CD34 cells rather than freshly isolated human BM cells [13]. Notably, colony assays were performed with BFU-E and CFU-E; however, miR-199b silencing did not affect erythroid proliferation (data not shown). Transcription factors like cKit and RUNX1, known regulators of HSCs and Sp1, a transcription factor that regulates myelopoiesis, are conservative targets of miR-199b [14–16]. Silencing of miR-199b in AML may in part lead to perturbed myelopoiesis via selective lineage specific dysregulation of key factors that direct myelopoiesis.

### Clinical implications of low-miR-199b in AML

**Fig. 2:**
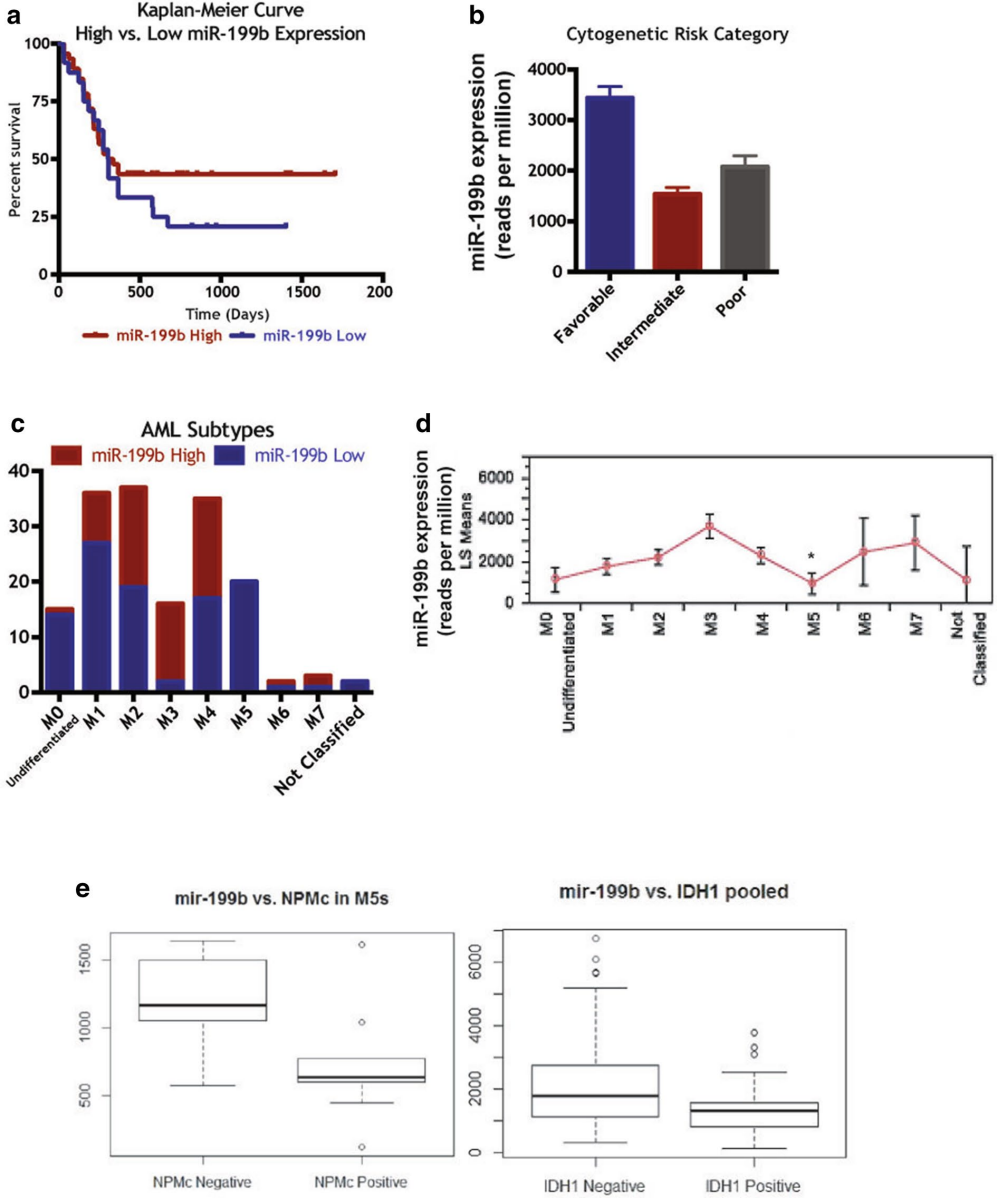
Clinical relevance of miR-199b-5p across AML subtypes. **a** Kaplan–Meier analysis of 166 AML patients from the TCGA database for high/low miR-199b expression and overall survival, p = 0.0164. **b** miR-199b cytogenetic risk for same patients set. **c** High/low miR-199b expression by FAB subtype in the same patients set. **d** Least squared means plot of 199b expression for different FAB subtypes using ANOVA (p < 0.0001). **e** miR-199b levels for n = 128 AML patients analyzed for miR-199b/NPM1 with n = 63 miR-199b-high samples, of which n = 2 were positive for NPM1 mutation, and n = 65 miR-199b-low samples of which n = 41 positive for NPM1 mutation (Fisher p value = 1.941e-08, odds ratio 19.6112) (*left panel*). miR-199b levels for n = 139 patients with n = 59 miR-199b-high samples (of which n = 5 positive for IDH1 mutation) whereas n = 80 were miR-199b-low samples with n = 25 positive for IDH1 mutation (Fisher p value = 0.01175, odds ratio 3.663005) (*right panel*)

In order to correlate miR-199b expression with clinical AML patient data to determine any prognostic role, we utilized the Cancer Genome Atlas (TCGA) to analyze the molecular and clinical characteristics of 166 AML cases. Kaplan–Meier curves for high and low expression of miR-199b along with the observed distribution of miRNA expression revealed the highly expressed group (>2000 reads per million miRNA mapped) showed significantly better survival outcome (Fig. 2a, p < 0.016, log rank test). In addition, miR-199b expression varied by cytogenetic risk category in the least squared mean plot, with significantly higher miR-199b expression in the favorable category compared to the intermediate and poor categories combined (Fig. 2b, p < 0.0001). Therefore, low expression of mir-199b predicts worse survival outcome and higher cytogenetic risk in AML.

Additionally, there was a significant difference in miR-199b expression across the AML FAB subtypes with particularly low numbers of miR-199b-low patients in the M5 subtype (Fig. 2c) as well as low expression in M5 subtype (Fig. 2d). Further, M5 subtype shows a poor prognosis with a one-year survival rate of only 25 %, compared with 51 % survival in the overall sample (p < 0.024 with a Chi squared goodness of fit test). With AML patients grouped into M5 versus other subtypes to look at the dichotomized miR-199b variable, we find all of the M5 patients have low miR-199b expression (p < 0.0001). Thus, it is possible low miR-199b expression is a potential reason for poor survival outcomes in this subtype, and targeted treatment may represent a personalized treatment regime for this subtype. Interestingly, most of the analyzed M5 AML patients were cytogenetically normal (CN-AML); however, these patients exhibited molecular mutations (Additional file 2: Table S1).

Next, we attempted to determine the potential cooperative role of these associated molecular abnormalities (FLT3-ITD, IDH–R140, -R132, -R172, activating Ras, NMPc) among miR-199b high and low AML patients. Significant correlations in terms of association and survival outcomes were observed for NPM1 and IDH1 mutations. Among n = 128 AML patients analyzed for miR-199b/NPM1, n = 63 were miR-199b-high samples and, of these, n = 2 were positive for NPM1 mutation. Importantly, of the n = 65 miR-199b-low samples, n = 41 were positive for NPM1 mutation (Fig. 2d, left panel, Fisher p value = 1.94e-08, odds ratio 19.6). For IDH1 mutation, among the n = 139 AML patients analyzed for miR-199b/IDH1, n = 59 were miR-199b-high samples (with n = 5 positive for IDH1 mutation) whereas n = 80 were miR-199b-low samples (with n = 25 positive for IDH1 mutation) (Fig. 2d, right panel, Fisher p value = 0.012, odds ratio 3.7). Having a better understanding of miR-199b’s regulation could provide insight into specific therapies for low-miR-199b AML cases. It is tempting to speculate that NPM1 or IDH1 mutation may play a cooperative role in miR-199b-low associated AML, which if proved can facilitate the exploration of combinatorial strategies with MLN9708, a recently discovered and therapeutically tested inhibitor of mutated NPM1 in AML [17] or AGI-6780 [18] for IDH2R140Q. Interestingly, miRNA expression analysis in AML patients expressing mutant NPM1 reveals significant alterations in expression profile of select miRNAs including miR-199a compared to AML with wild type NPM1 [19].

**Fig. 3:**
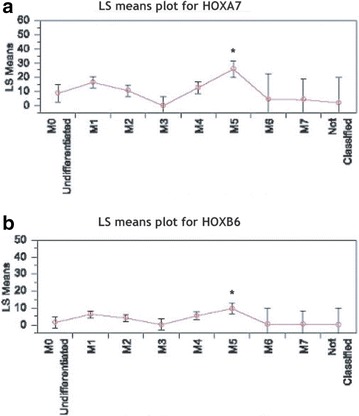
Expression of HoxA7 and HoxB6 inversely correlates with miR-199b levels in AML with FAB M5 subtype. **a** Least square (LS) means plots of conserved miR-199b target HOXA7 (ANOVA p < 0.0001) versus different AML FAB categories. ANOVA *p < 0.0001 **b** Least square (LS) means plot of conserved miR-199b target HOXB6 (ANOVA p < 0.0017) versus different AML FAB categories. ANOVA *p < 0.0017

Next, in order to identify the molecular mechanism that could mediate the poor survival outcome and subtype specificity associated with low miR-199b expression, we queried gene expression data for co-expressed genes. A large number of genes were significantly correlated or anti-correlated with miR-199b expression, with the most significant negatively correlated genes listed (Additional file 3: Table S2). Included in this list are a large number of transcription factors in the HOX family which have been previously implicated in leukemia [20], and two of which, HOXA7 and HOXB6, are known targets of miR-199b. Hox family of transcription factors have been shown to play critical regulatory roles during hematopoiesis and their aberrant expression results in hematological malignancies [21]. To see if the pattern of expression by subtype is reversed (since these genes are anti-correlated or transcriptionally repressed by 199b), we performed an ANOVA of 199b expression by subtype and found that indeed the expression of these genes is highest in the M5 subtype, where 199b expression is the lowest (see Fig. 3a, b). Importantly, HOXA7 and HOXB6 expression was significantly elevated in miR-199b-low AML samples with FAB-M5 subtype (Fig. 3a, b), which increases the possibilities of important transcription factors coming in the regulatory ambit of miR-199b in HSCs.

### Epigenetic regulation of miR-199b

Due to miR-199b significantly correlating with M5 subtype, M5 cell line THP-1 was utilized to determine the epigenetic regulation of miR-199b in AML. Epigenetic alterations such as histone modifications and DNA methylation have been shown to deregulate miRNA expression. One example is miR-193a being modified via HDACs and DNA methyltransferases (DNMTs) associated with methylation through AML1/ETO recruitment of these factors [22]. Therefore, utilizing HDAC inhibitors and demethylating agents can identify methylation status and histone modifications of miR-199b’s promoter and potentially be utilized as therapeutics in low-miR-199b AML cases.

**Fig. 4:**
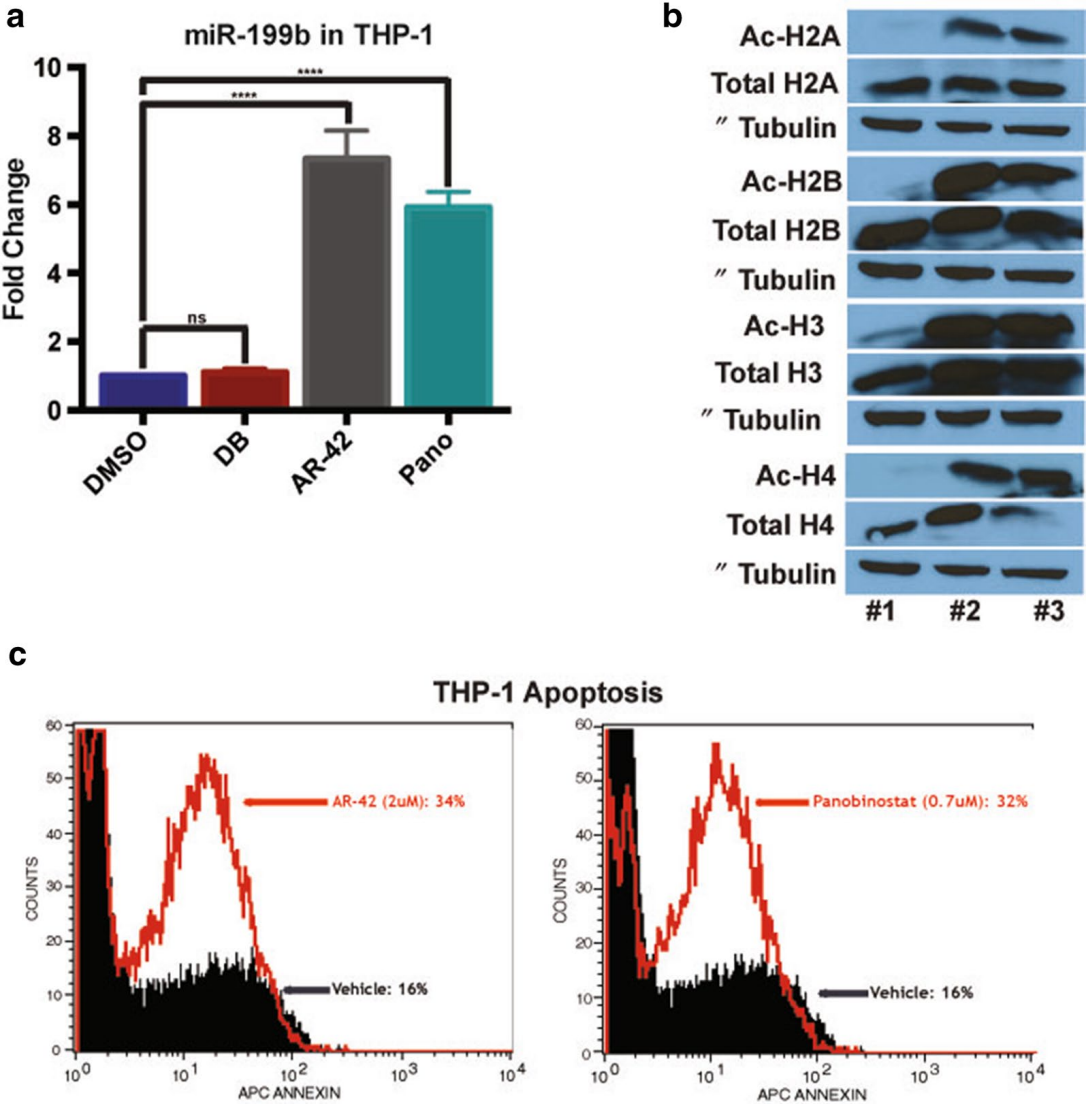
HDAC inhibitors restore miR-199b-5p expression in THP-1 cells to induce apoptosis. **a** miR-199b expression in THP-1 cells upon 24 h treatment with vehicle (DMSO), Decitabine (DB, 5uM), AR-42 (2uM), or Panobinostat (0.7uM) ****p < 0.0001. **b** Western blot for THP-1 cells treated with DMSO [27], Panobinostat [28] or AR-42 [29] blotted for acetylated (Ac) and total histones H2A, H2B, H3 and H4. **c** Apoptosis via Annexin V staining for the AR-42 and Panobinostat treated cells compared to DMSO treatment

Treatment of THP-1 cells (24 h) with AR-42 (HDAC inhibitor), Panobinostat (Pano, HDAC inhibitor), or Decitabine (DB, demethylating agent) showed miR-199b expression was significantly elevated upon AR-42 and Pano treatment (p < 0.0002 and p < 0.0001 respectively) (Fig. 4a). To determine the efficacy of these HDAC inhibitors, acetylation of histones were analyzed via Western blot analysis (Fig. 4b). Further, apoptosis via Annexin V staining was analyzed in AR-42 and Pano treated cells to determine the functionality of these treatments and showed a marked increase in apoptosis in both treatments (Fig. 4c). Thus, epigenetic regulation of miR-199b appears to be via histone modification.

Taken together, our results demonstrate in vitro loss of miR-199b can lead to myeloproliferation and HSC proliferation while HDAC inhibitors (AR-42 or Pano) can recover miR-199b expression and promote apoptosis in leukemic cells. Both AR-42 and Pano are both clinically relevant as both are in clinical trials. For example, AR-42 is in one clinical trial in combination with DB for patients with AML (ClinicalTrials.gov identifier: NCT01798901). One supporting study for this trial may be when AML cells were treated with AR-42; it raises miR-29b expression and primes the cells for DB treatment to induce apoptosis [23]. A clinical trial is currently pairing Pano with routine therapies of idarubicin and cytarabine in AML patients over the age of 65 (ClinicalTrials.gov identifier: NCT00840346). Both of these inhibitors could be a mode of therapy for AML patients with low-miR-199b.

### miR-199b-low transplanted mouse

**Fig. 5:**
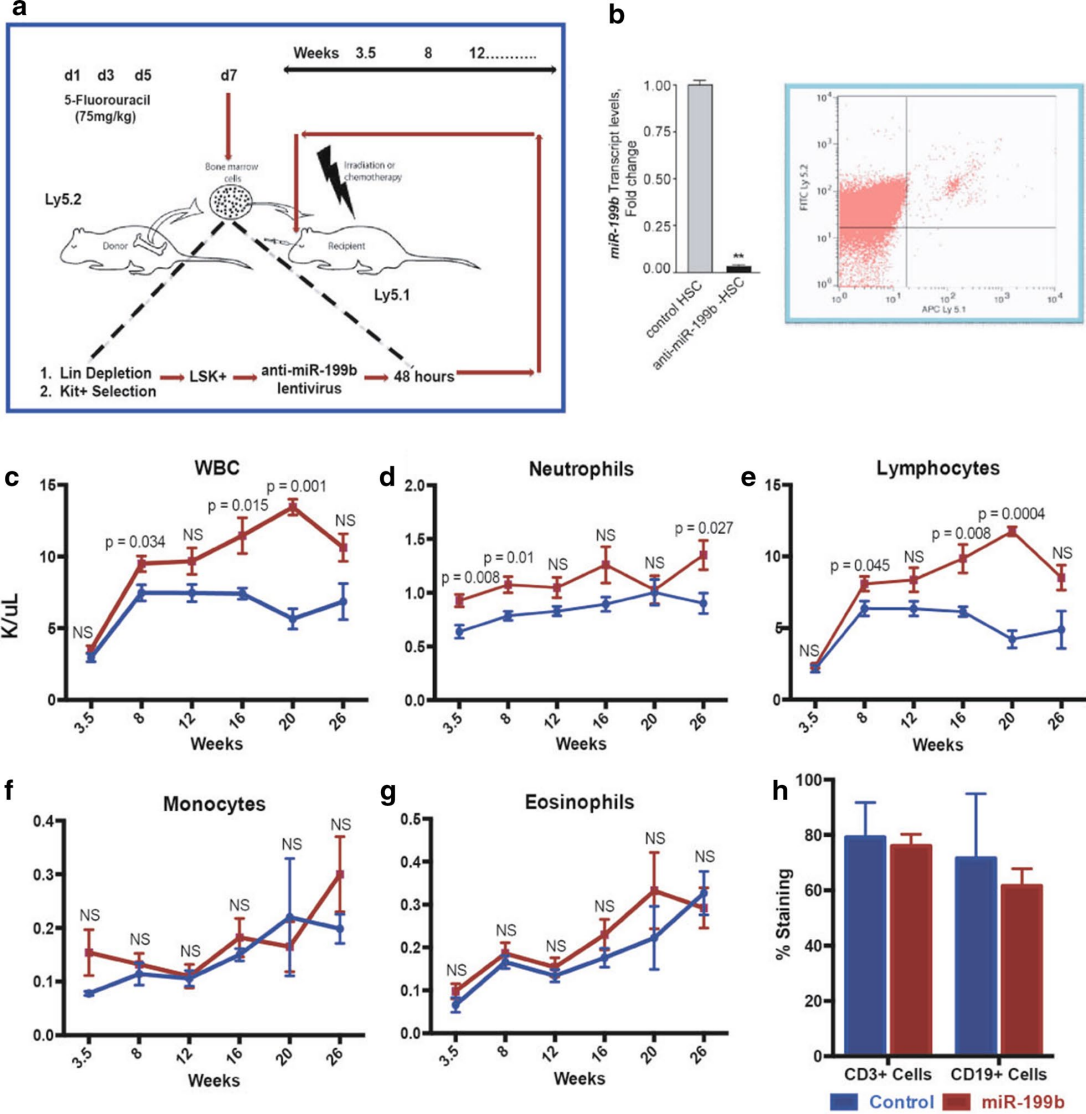
miR-199b silencing in vivo via a transduce and transplant approach. **a** Bone marrow transplant (BMT) approach schematic to delete miR-199b in hematopoietic cells. Mice were treated with 5-flourouracil, LSK (Lin^−^Sca^+^Kit^+^) cells were obtained and silenced with an anti-miR-199b lentivirus. These cells were then transplanted in irradiated Ly5.1 mice. **b** *Left panel*: Lentiviral particles for anti-miR-199b-5p and control vector were used to transduce mouse HSCs. Silencing of miR-199b levels were confirmed in HSCs post transduction via RTqPCR. *Right panel*: Engraftment efficiencies post BMT were measured using staining for Ly5.2 versus Ly5.1 via flow cytometry. A representative flow cytometry data is shown from the analysis. **c**–**g** Peripheral blood analysis for control and low-miR-199b transplant mice (n = 5). **h** Flow cytometry of peripheral blood B (CD19 staining) and T (CD3 staining) cells in control and low-miR-199b transplant mice at 27 weeks

To further understand the hematopathological consequences of decreased miR-199b in the onset and development of myeloid leukemia, we employed a transduce/transplant mouse model (Fig. 5a). The Lin^−^Sca-1^+^Kit^+^ (LSK) population was isolated from Ly5.2 C57Bl6 mice; transduced with anti-miR-199b or control lentivirus, and, using RT-qPCR, we confirmed that miR-199b was indeed much lower in these LSK cells (Fig. 5b, left panel). The transplanted LSK cells into Ly5.1 recipient mice post sub-lethal irradiation.

Once the transplantation’s efficacy was confirmed by Ly5.1 and Ly5.2 staining of peripheral blood (PB) via flow cytometry (Fig. 3b, right panel) we hoped to observe leukemia in these mice by performing blood analysis. This analysis began at 3.5 weeks post-transplantation and continued every 4–6 weeks afterward to assess the complete blood count and observe the mice for any potential distress (Fig. 5c–g). Short-term (3.5 weeks) PB analysis determined loss of miR-199b results in significant increase of neutrophils that was significantly sustained until 8 weeks post-transplantation (Fig. 5d). By 20 weeks post-transplantation the neutrophil counts recovered completely until they rose significantly again at 26 weeks. Early on, this indicated loss of miR-199b expression in HSCs could have leukemic potential but the recovery and subsequent increase through the 26 weeks indicate that on a myeloid leukemia standpoint, miR-199b may not itself be leukemogenic.

Again, it is possible miR-199b works cooperatively with other mutations, as we suggested previously with IDH1 and NPM1 mutations. Since miR-199b expression is highest in CD33^+^ cells during steady state hematopoiesis, perhaps the loss of miR-199b needs to be at the CD33^+^ stage instead of the CD34^+^ stage to induce myeloid leukemia. Nonetheless, we chose to assess the complete blood count to determine any additional patterns in the blood profile since miR-199b was silenced in the HSCs and could be affecting all blood types. We saw no significant changes in monocytes or eosinophils across the time course in the mice (Fig. 5f, g). Surprisingly, the whole white blood cell (WBC) count saw significant increases at weeks 8, 16, and 20 where it peaked before it was no longer significant at week 26 (Fig. 5c).

These results interestingly, were mirrored by the lymphocyte counts, which do contribute a large number to the WBC count (Fig. 5e). From this we can conclude that there may be either an immune response to the low miR-199b or low miR-199b may play an important role in not only myelopoiesis but also lymphopoiesis. An interesting avenue to investigate further may be miR-199b’s expression in different types of lymphocytic leukemia or lymphoma to see if miR-199b is implicated in these forms of blood cancers. miR-199b has yet to be linked to any lymphocytic leukemias or lymphomas but there is a report that links low miR-199a expression in primary CNS lymphoma cases [24] therefore with the sequence homology between miR-199a and miR-199b, miR-199b could be dysregulated in lymphomas as well and should be assessed via the TCGA database. At 27 weeks we chose to follow-up with flow cytometry for B and T cell markers to see if a specific cell type was increasing the high lymphocyte counts. Unfortunately, at this point, there were no significant difference between control and low-miR-199b mice for the B or T cells (Fig. 5h). This could have been due to the fact that the lymphocyte numbers peaked at week 20.

## Conclusions

More targeted studies are needed to determine how low miR-199b in HSCs leads to a myeloproliferative phenotype. In summary, it appears miR-199b plays an important regulatory role in differentiation and HDAC inhibitors are able to rescue miR-199b expression. MiR-199b appears to be a promising prognostic marker for FAB-M5-AML and CN-AML with NPM1 mutation. This association is correlated with relatively poor survival; furthermore, FAB-M5-AML also correlates with heightened HOXA7 and HOXB6 expression, two transcription factors known to be targets of miR-199b. Though our in vivo transplant mouse model showed only transient hematopoietic perturbations, it is possible miR-199b works cooperatively with these other mutations as part of a two-hit model leading to AML. Further studies are underway to examine the miR-199b’s cooperativity, possibly with NPM1, and how this mechanistically results in myeloproliferation.

## Methods

### Primary cell and RNA samples

Primary human bone marrow (BM) CD34^+^, BM CD33^+^, PB monocytes, PB eosinophils, and PB basophils RNA was obtained from stem cell technologies for transcript analysis.

### miRNA isolation, cDNA synthesis, and RT-qPCR

In order to determine the relative expression of miR-199b, miRNA was isolated from cells using miRCURY RNA Isolation Kit (Exiqon) following the manufacturer’s instructions. cDNA was made utilizing the Universal cDNA Synthesis Kit II (Exiqon) for miRNA. RT-qPCR was performed using the miRCURY LNA Universal RT microRNA PCR system (Exiqon) and 2X SYBR Green master mix (SYBR Green/Fluorescein, cat #330513, SA Biosciences) on the iQ-Cycler (Bio-Rad) with the following primers (Exiqon) for miRNA analyses: hsa-miR-199b-5p LNA™ PCR primer set, UniRT (Mature sequence: CCCAGUGUUUAGACUAUCUGUUC) and Reference primer set: U6 snRNA (hsa, mmu, rno).

### CD34 cell culture, lentiviral transduction, CFC assay and HSC proliferation assay

In order to assess colony forming capabilities in normal CD34^+^ cells with low-miR-199b we purchased CD34^pos^ cells (Stem Cell Tech) and expanded in StemSpan SFEM (StemCell Tech) containing FLT3 (100 ng/mL), IL-3, IL-6 (10 ng/mL each), SCF (100 ng/mL) and 1 % pen/strep (Invitrogen). Lentiviral particles for hsa-miR-199b were generated by cotransfecting pEZX-AM03 vector expressing anti-miR-199b-5p (from H1 promoter) and mCherry fluorescent protein (CMV promoter) into 293T cells with the Lenti-Pac HIV packaging mix. Lentivirus products were titrated by quantitative RT-PCR, which determines the copy number of viral genomic RNA (titer = 3.3 × 10^10^ copies/mL). Anti-miR-199b lentivirus particles at a multiplicity of infection (MOI) between 10 and 15 were added to the CD34^+^ cells at 37 °C. For controls, lentivirus particles expressing mCherry were used at similar MOIs. The cells were infected for 15 h and then recovered in culture medium. Integration sites were verified by ligation mediated-PCR (LM-PCR) and sequencing. Significant silencing (95 %) of miR-199b expression was confirmed via RT-qPCR analysis. For colony forming assays, cell suspensions of 2 × 10^5^ cells/mL were prepared and 0.4 mL of cells was added to 4 mL MethoCult medium containing GM-CSF, SCF and IL-3. A 16-gauge blunt-end needle attached to a 5 mL syringe was used to dispense the cells and MethoCult medium into culture dishes (n = 3 in duplicates). CFU-GM colonies were counted with bright field and fluorescent microscopy 16 days after the cells were plated.

### The cancer genome atlas analysis

When analyzing miR-199b levels in AML patient samples, level 3 gene expression, microRNA expression, and clinical data for 200 AML patients were downloaded from the data portal for the Cancer Genome Atlas Project (TCGA, http://www.cancergenome.nih.gov). 187 of these samples had IlluminaGA RNASeq data for microRNA expression, 179 samples had IlluminaGA RNASeq data for gene expression, and 166 samples had non-missing data for survival time, gene, and microRNA expression and were used in subsequent analyses. All analyses were performed in R version 3.0.1 using the RStudio environment.

Box plots and t tests were performed for the association between high and low miRNA expression (dichotomized 2000 reads per miRNA mapped for 199b) and gene expression for candidate genes using standard R packages, with log transformations performed for expression variables due to lack of normality of raw variables. In fitting a Cox proportional hazards model to the continuous miRNA expression level, a significant model is found (p < 0.021; check proportional hazards assumption) with a risk ratio of 0.3 (0.10, 0.84), reciprocal 3.32, over the range of miRNA expression seen in this cohort. Kaplan–Meier curves were performed using the survival package in R (survfit and survdiff functions), with the log rank *p* value used for testing difference in survival curves over strata. Tests of association between dichotomized microRNA expression and gene expression variables were performed using a Fisher’s Exact test (fisher.test function in R).

### Ethics, consent, and permissions

The TCGA studies were performed in accordance with the principles of the Declaration of Helsinki (http://cancergenome.nih.gov/newsevents/newsannouncements/TCGA_AML_press_release_2013) [25].

#### Cell culture

THP-1 cell line was cultured in RPMI-1640 Medium with 0.05 mM 2-mercaptoethanol, 10 % fetal bovine serum and 1× penicillin, streptomycin, fungizone.

#### Inhibitors

THP-1 cells were treated with vehicle (DMSO), 5 μM Decitabine (DB), 2 μM AR-42, or 0.7 μM Panobinostat for 24 h for miR-199b-5p expression studies, apoptosis analysis via Annexin V staining, and protein expression via Western blot analysis.

#### Western blot

THP-1 cells with indicated treatments were lysed in M-PER mammalian protein extraction lysis buffer (Thermo Scientific, Cat #78501) containing Halt protease and phosphatase inhibitor cocktail (Thermo Scientific, Cat #78442) and cleared lysates were assayed for protein content, denatured, electrophoresed, transferred to PVDF membranes, blocked and probed with the indicated antibodies. Primary antibodies for both acetylated and total Histones H2A, H2B, H3, and H4 as well as beta-tubulin were obtained from cell signaling. HRP-conjugated antibodies and ECL reagents were as described previously [26].

#### Annexin V staining via flow cytometry

To analyze cell death, cells were stained with Annexin V (BD Pharmingen) and Propidium Iodide (invitrogen). Prior to staining, cells were washed with PBS and resuspended in 1× Annexin V binding buffer (BD Biosciences) and staining was performed by manufacturers instructions. After incubation, samples were analyzed via flow cytometry on the FACS Caliber (BD Biosciences).

#### Isolation and transduction of HSC with anti-miR-199b

In order to assess the effect of low-miR-199b in vivo, LSK cells were taken from donor mice and transduced with anti-miR-199b before being transplanted into recipient mice. To achieve this bone marrow from C57BL6/J (Ly5.2) mice was obtained for transduction of HSCs. Prior to extracting bone marrow, mice received intraperitoneal injections on days 1, 3, and 5 with 5-fluorouracil (75 mg/kg). On day 7 cells were extracted, the LSK (Lin^−^Sca^+^Kit^+^) cells were enriched via bead selection kits (Stem Cell Technologies) and maintained in culture conditions. Anti-miR-199b lentivirus particles at a MOI between 10 and 15 were added to the cells at 37 °C on Retronectin coated plates per manufacturers instructions. For controls, lentivirus particles expressing mCherry were used at similar MOIs. The cells were infected for 48 h and then recovered in culture medium before transplantation. Significant silencing (95 %) of miR-199b expression was confirmed via RT-qPCR analysis.

#### Bone marrow transplantations (BMT)

Control and anti-miR-199b transduced donor bone marrow (methods mentioned above) cells at 5 × 10^5^ cells were transplanted via retro-orbital injection into irradiated B6 Ptprca (Ly5.1) recipients who underwent radiation (450 cGy at 4 and 1 h before transplantation) to deplete their bone marrow. To confirm transplantation was effective, Ly5.1 and Ly5.2 staining was analyzed on PB via flow cytometry (see below).

#### Flow cytometry

Upon red cell lysis, cells were incubated with Ly5.1 and Ly5.2 antibodies (BD Biosciences) for 30 min to determine transplant efficiency. For B (CD19) and T (CD3) cell staining, similar methods were employed. Following incubation, cells were washed and re-suspended in 500 μL of PBS and analyzed on flow using FACScalibur (BD Biosciences).

#### Procyte blood analysis

A PB profile of transplanted miR-199b-low mice was completed at 3.5, 8, 12, 16, 20 and 26 weeks post-transplantation. Mice were first anesthetized with isoflurane. Blood was collected using retro-orbital eye bleeding with heparinized capillary tubes to obtain 50–75 μL of blood. This blood was then analyzed on the ProCyte Dx Hematology Analyzer (IDEXX) for a complete blood count profile output.

## Additional files

**Additional file 1: Figure S1.** miR-199b-5p targets HIF-1 alpha. Transcript levels of three predicted targets of miR-199b were tested via RT-qPCR in miR-199b silenced CD34 cells and HIF-1a levels were significantly increased by anti-miR-199b.

**Additional file 2: Table S1.** Clinical and molecular characteristics of AML patients with FAB-M5 subtype.

**Additional file 3: Table S2.** List of genes most significantly negatively correlated with expression of miR-199b in the AML data set (p-value calculated from a Fisher’s transformed Pearson correlation coefficient). A large number of HOX family transcription factors are found in this list, two of which, HOXA7 and HOXB6, are known targets of miR-199b.
