# Supplementary figures and images for: miR-199b, a novel tumor suppressor miRNA in acute myeloid leukemia with prognostic implications
Source: Exp Hematol Oncol. 2016 Feb 3;5:4. doi: 10.1186/s40164-016-0033-6 (PMC4740997; doi:10.1186/s40164-016-0033-6)

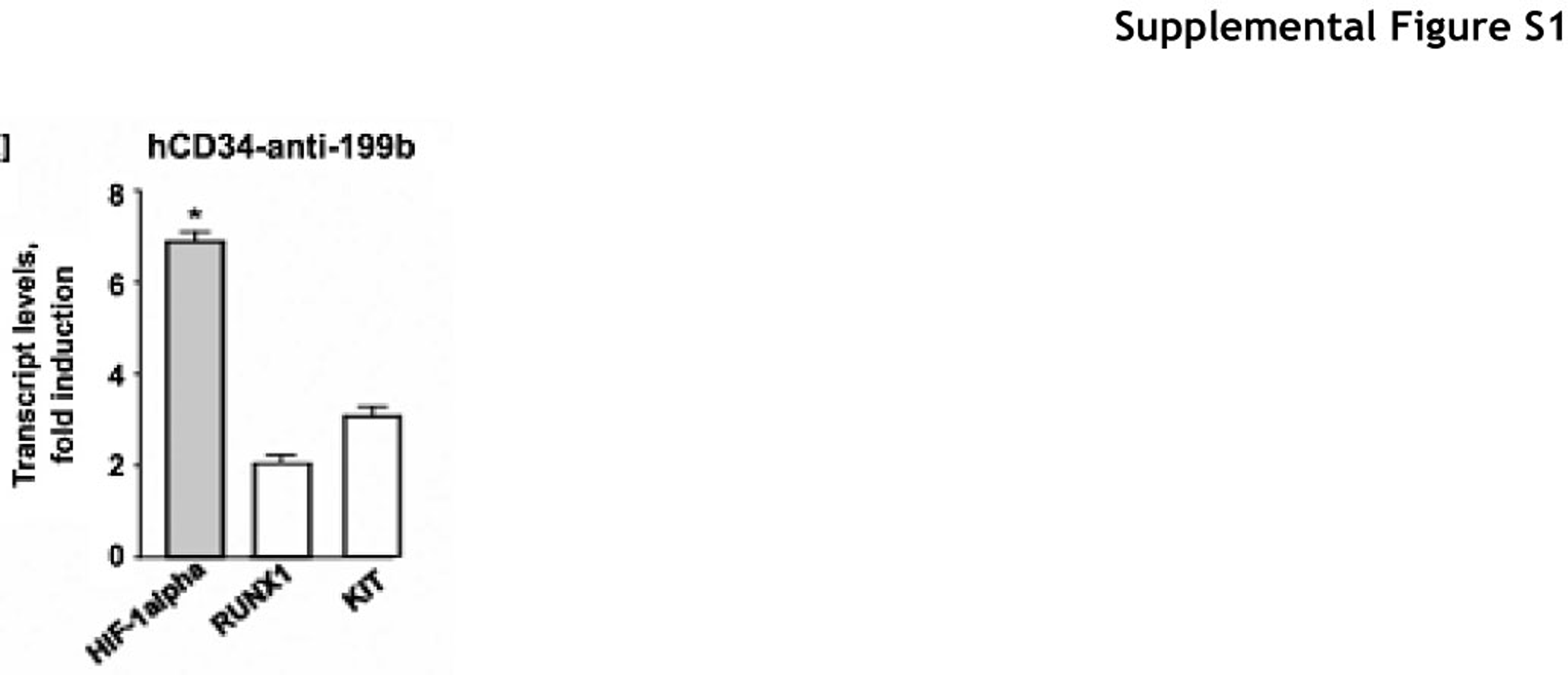

Supplement: Supplementary file 1 — Additional file 1: Figure S1. miR-199b-5p targets HIF-1 alpha. Transcript levels of three predicted targets of miR-199b were tested via RT-qPCR in miR-199b silenced CD34 cells and HIF-1a levels were significantly increased by anti-miR-199b. [file 40164_2016_33_MOESM1_ESM.tif]
