# Supplementary material for: miR-199b, a novel tumor suppressor miRNA in acute myeloid leukemia with prognostic implications
Source: Exp Hematol Oncol. 2016 Feb 3;5:4. doi: 10.1186/s40164-016-0033-6 (PMC4740997; doi:10.1186/s40164-016-0033-6)
Supplement: Supplementary file 2 — Additional file 2: Table S1. Clinical and molecular characteristics of AML patients with FAB-M5 subtype. [file 40164_2016_33_MOESM2_ESM.pdf]

Supplemental Table S1
Clinical and Molecular Characteristics with FAB M5 Subtype

TCGA ID#	Age at Diagnosis	Vital Status	Cytogenetic Abnormality	FLT3	IDH1 R132	IDH1 R140	IDH1 R172	Activating RAS	NPMc
TCGA-AB-2824	45	DECEASED	Normal	-	-	-	-	-	+
TCGA-AB-2825	31	DECEASED	Normal	+	-	-	-	-	+
TCGA-AB-2832	60	DECEASED	Normal	-	-	-	-	-	-
TCGA-AB-2835	48	LIVING	Normal	-	-	-	-	-	+
TCGA-AB-2837	66	DECEASED	Normal	-	-	-	-	-	+
TCGA-AB-2851	66	DECEASED	Normal	+	-	-	-	-	-
TCGA-AB-2861	76	DECEASED	Normal, Trisomy 8	-	-	-	-	-	+
TCGA-AB-2873	51	LIVING	Normal	-	-	-	-	-	-
TCGA-AB-2883	60	LIVING	Normal, Trisomy 8	-	-	-	-	-	-
TCGA-AB-2893	45	DECEASED	N/A	-	-	-	-	+	-
TCGA-AB-2894	50	DECEASED	Normal, del (7q) / 7q-|t (9;11)	-	-	-	-	-	-
TCGA-AB-2910	61	DECEASED	Normal	+	-	-	-	-	-
TCGA-AB-2925	57	DECEASED	Normal	+	-	-	-	-	+
TCGA-AB-2932	62	LIVING	Normal	-	N/A	-	-	-	+
TCGA-AB-2956	61	DECEASED	Normal, t (9;11)	-	-	-	-	-	-
TCGA-AB-2969	55	LIVING	Normal	+	+	-	-	-	+
TCGA-AB-2981	35	LIVING	Normal	+	+	-	-	-	+
TCGA-AB-2985	81	DECEASED	Normal	-	-	-	-	+	-
TCGA-AB-2987	75	DECEASED	Normal, del (7q) / 7q-	-	-	-	-	+	+
TCGA-AB-3005	45	LIVING	Normal	N/A	-	-	-	-	-
