# Supplementary material for: miR-199b, a novel tumor suppressor miRNA in acute myeloid leukemia with prognostic implications
Source: Exp Hematol Oncol. 2016 Feb 3;5:4. doi: 10.1186/s40164-016-0033-6 (PMC4740997; doi:10.1186/s40164-016-0033-6)
Supplement: Supplementary file 3 — Additional file 3: Table S2. List of genes most significantly negatively correlated with expression of miR-199b in the AML data set (p-value calculated from a Fisher’s transformed Pearson correlation coefficient). A large number of HOX family transcription factors are found in this list, two of which, HOXA7 and HOXB6, are known targets of miR-199b. [file 40164_2016_33_MOESM3_ESM.pdf]

Supplementary Table S2

p-value	Gene	Entrez Gene Name	Location	Type(s)
1.10E-11	C22orf26	chromosome 22 open reading frame 26	unknown	other
2.37E-11	HOXA3	homeobox A3	Nucleus	transcription regulator
4.36E-11	CPNE8	copine VIII	unknown	other
1.30E-10	HOXA6	homeobox A6	Nucleus	transcription regulator
2.31E-10	HOXB3	homeobox B3	Nucleus	transcription regulator
4.67E-10	LOC100271722	uncharacterized LOC100271722	unknown	other
5.11E-10	HOXB6	homeobox B6	Nucleus	transcription regulator
8.84E-10	SARM1	sterile alpha and TIR motif containing 1	Plasma Membrane	transmembrane receptor
9.67E-10	HOXA4	homeobox A4	Nucleus	transcription regulator
1.29E-09	SLC46A1	solute carrier family 46 (folate transporter), member 1	Plasma Membrane	transporter
1.57E-09	RIPK3	receptor-interacting serine-threonine kinase 3	Plasma Membrane	kinase
1.85E-09	HOXA7	homeobox A7	Nucleus	transcription regulator
2.70E-09	HOXA5	homeobox A5	Nucleus	transcription regulator
4.36E-09	HOXA9	homeobox A9	Nucleus	transcription regulator
4.94E-09	TMCO4	transmembrane and coiled-coil domains 4	unknown	other
6.81E-09	HOXB4	homeobox B4	Nucleus	transcription regulator
7.16E-09	HOXB5	homeobox B5	Nucleus	transcription regulator
1.04E-08	HOXA10	homeobox A10	Nucleus	transcription regulator
1.05E-08	RMND5B	required for meiotic nuclear division 5 homolog B (S. cerevisiae)	unknown	other
1.58E-08	WDR91	WD repeat domain 91	unknown	other
1.59E-08	HOXB2	homeobox B2	Nucleus	transcription regulator
1.63E-08	TCTA	T-cell leukemia translocation altered	unknown	other
1.74E-08	ACBD4	acyl-CoA binding domain containing 4	unknown	other
3.40E-08	IQCE	IQ motif containing E	Cytoplasm	other
4.58E-08	TTYH3	tweety homolog 3 (Drosophila)	Plasma Membrane	ion channel
8.05E-08	AIFM2	apoptosis-inducing factor, mitochondrion-associated, 2	Cytoplasm	enzyme
9.70E-08	PARP3	poly (ADP-ribose) polymerase family, member 3	Nucleus	enzyme
9.95E-08	MVP	major vault protein	Nucleus	other
1.20E-07	DUSP28	dual specificity phosphatase 28	unknown	enzyme
1.25E-07	LTB4R	leukotriene B4 receptor	Plasma Membrane	G-protein coupled receptor
1.38E-07	FNBP1	formin binding protein 1	Nucleus	enzyme
1.41E-07	AGXT2L2	alanine-glyoxylate aminotransferase 2-like 2	Cytoplasm	enzyme
1.41E-07	STK10	serine/threonine kinase 10	Cytoplasm	kinase
1.47E-07	MEIS1	Meis homeobox 1	Nucleus	transcription regulator
1.66E-07	CDK18	cyclin-dependent kinase 18	Cytoplasm	kinase
1.72E-07	STK32C	serine/threonine kinase 32C	unknown	kinase
2.08E-07	HOXA2	homeobox A2	Nucleus	transcription regulator
2.32E-07	CLIP4	CAP-GLY domain containing linker protein family, member 4	unknown	other
2.33E-07	CCDC97	coiled-coil domain containing 97	unknown	other
